# Liver fibrosis is associated with carotid atherosclerosis in patients with liver biopsy-proven nonalcoholic fatty liver disease

**DOI:** 10.1038/s41598-021-95581-8

**Published:** 2021-08-05

**Authors:** Taeang Arai, Masanori Atsukawa, Akihito Tsubota, Keizo Kato, Hiroshi Abe, Hirotaka Ono, Tadamichi Kawano, Yuji Yoshida, Tomohide Tanabe, Tomomi Okubo, Korenobu Hayama, Ai Nakagawa-Iwashita, Norio Itokawa, Chisa Kondo, Keiko Kaneko, Naoya Emoto, Mototsugu Nagao, Kyoko Inagaki, Izumi Fukuda, Hitoshi Sugihara, Katsuhiko Iwakiri

**Affiliations:** 1 Division of Gastroenterology and Hepatology, Nippon Medical School, 1-1-5, Sendagi, Bunkyo-ku, Tokyo, 113-8603 Japan; 2 Core Research Facilities for Basic Science, Research Center for Medical Sciences, The Jikei University School of Medicine, 3-25-8, Nishi-Shimbashi, Minato-ku, Tokyo, 105-8461 Japan; 3 Division of Gastroenterology and Hepatology, Shinmatusdo Central General Hospital, 1-380, Shin-matsudo, Matsudo-shi, Chiba, 270-0034 Japan; 4 Division of Gastroenterology, Nippon Medical School Chiba Hokusoh Hospital, 1715 Kamagari, Inzai-shi, Chiba, 270-1694 Japan; 5 Division of Endocrinology, Nippon Medical School Chiba Hokusoh Hospital, 1715 Kamagari, Inzai-shi, Chiba, 270-1694 Japan; 6 Division of Endocrinology, Diabetes and Metabolism, Nippon Medical School, 1-1-5, Sendagi, Bunkyo-ku,, Tokyo, 113-8603 Japan

**Keywords:** Cardiology, Gastroenterology, Risk factors

## Abstract

Nonalcoholic fatty liver disease (NAFLD) is related to subclinical atherosclerosis. However, whether the severity of the disease (or which histopathological component) is associated with subclinical atherosclerosis remains controversial. This study aimed to investigate the association between the histopathological severity of NAFLD and carotid intima-media thickness (CIMT) in Japanese patients with liver biopsy-proven NAFLD. Maximum-CIMT (max-CIMT) was measured as an index of carotid atherosclerosis in 195 biopsy-proven NAFLD patients. A significant association was observed between the severity of fibrosis (but not steatosis, inflammation, and ballooning) and max-CIMT. Older age, male gender, hypertension, and advanced fibrosis were independently linked to max-CIMT ≥ 1.2 mm. The prevalence of max-CIMT ≥ 1.2 mm was significantly higher in the advanced fibrosis group than in the non-advanced fibrosis group (75.4% versus 44.0%; *p* < 0.01). Non-invasive liver fibrosis markers and scoring systems, including fibrosis-4 index, NAFLD fibrosis score, hyaluronic acid, and Wisteria floribunda agglutinin positive Mac-2-binding protein, demonstrated that the diagnostic performance for max-CIMT ≥ 1.2 mm was similar to that of biopsy-based fibrosis staging. In conclusion, advanced fibrosis is significantly and independently associated with high-risk CIMT. Non-invasive fibrosis markers and scoring systems could help estimate the risk of atherosclerosis progression in patients with NAFLD.

## Introduction

Nonalcoholic fatty liver disease (NAFLD) is the most common cause of chronic liver disease worldwide. The global prevalence of NAFLD has been increasing and has reached approximately 25% of the adult population^1^. Among patients with NAFLD, liver fibrosis often occurs and progresses from steatohepatitis to liver cirrhosis, and other complications, including liver failure and hepatocellular carcinoma, also occurs^2,3^. Furthermore, as NAFLD is considered to be the hepatic phenotype of metabolic syndrome with multi-organ involvement, patients with NAFLD frequently have impaired cardiovascular, endocrine, renal, and bone systems. It has been recently suggested that NAFLD is a key driver of multi-organ syndrome, including cardiovascular disease (CVD), atherosclerosis, chronic kidney disease, type 2 diabetes mellitus, and osteoporosis^4,5^. From this viewpoint, hepatic involvement is just one aspect of NAFLD, and attention should be paid to other organ disorders in addition to the liver. Indeed, the most common causes of mortality among patients with NAFLD are CVD, followed by extrahepatic malignancies, and then liver-related complications^6–8^.

It is worth noting that NAFLD increases the risk of subclinical atherosclerosis and coronary artery calcification, independent of conventional risk factors, such as older age, type 2 diabetes mellitus, hypertension, and dyslipidemia^9–11^. Moreover, advanced liver fibrosis has been reported to have a substantial effect on the incidence and mortality of CVD in patients with NAFLD^8,12^. Some studies demonstrated that liver fibrosis assessed by non-invasive fibrosis scoring systems such as fibrosis-4 (FIB-4) index^13^ and NAFLD fibrosis score (NFS)^13–15^, could be associated with carotid intima-media thickness (CIMT), which is a risk index of CVD^16,17^. Hyaluronic acid^18^ and Wisteria floribunda agglutinin positive Mac-2-binding protein (WFA^+^-M2BP)^19–21^, which have been reported as useful liver fibrosis markers in NAFLD, have recently attracted attention as indicators of early atherosclerosis^22–24^, albeit they remain to be investigated in association with carotid atherosclerosis in NAFLD. Meanwhile, other studies have reported that advanced fibrosis evaluated via transient elastography was not associated with CIMT^25^. Therefore, the association between the severity of liver fibrosis and carotid atherosclerosis remains controversial. We have recently reported that advanced fibrosis is associated with brachial-ankle pulse wave velocity independent of conventional risk factors in 153 Japanese patients with liver biopsy-proven NAFLD^26^. However, there have been only two studies that verified the association between the histopathological severity of liver fibrosis and CIMT in patients with liver biopsy-proven NAFLD^27,28^. Moreover, CIMT is different in different races^29,30^, and both of these studies were conducted in Italy^27,28^. This study aimed to investigate the association between the histopathological severity of NAFLD and CIMT in Japanese patients with liver biopsy-proven NAFLD. We also asked whether non-invasive fibrosis markers and scoring systems could be useful for predicting the risk value of CIMT.

## Results

### Patients

Of the 357 patients with suspected NAFLD, 195 biopsy-proven NAFLD patients underwent carotid artery ultrasonography within 3 months of liver biopsy and included in the study (Supplementary Fig. 1). Baseline characteristics of the 195 patients with liver biopsy-proven NAFLD are presented in Table 1. There were 90 males and 105 females, with a median age of 59 years (range, 18–84 years). The prevalence of hypertension was 51.8% (101/195), and that of type 2 diabetes mellitus was 40.0% (78/195). Of the 78 NAFLD patients with type 2 diabetes mellitus, 64 (82.1%) had been receiving diabetes medications. On the pathological examination of liver biopsy specimens, the fibrosis stage was F0 for 29 (14.9%) patients, F1 for 62 (31.8%), F2 for 43 (22.1%), F3 for 49 (25.1%), and F4 for 12 (6.2%). Advanced fibrosis (F3–4) was present in 61 (31.3%) patients. The median maximum-CIMT (max-CIMT) value was 1.2 mm (range, 0.4–4.4 mm). The prevalence of max-CIMT ≥ 1.2 mm was 53.8% (105/195).

**Table 1:** Baseline characteristics of the 195 patients.

Factors	n = 195
Age (year)	59 (18–84)
Gender (male)	90 (46.2%)
BMI (kg/m^2^)	28.5 (18.1–44.9)
Platelets (× 10^4^/mm^3^)	19.0 (5.1–41.1)
AST (U/L)	55 (18–182)
ALT (U/L)	70 (10–401)
γ-GTP (U/L)	61 (14–391)
Serum albumin (g/dL)	4.0 (2.3–5.2)
eGFR (mL/min/1.73m^2^)	76 (29–145)
Prothrombin time (%)	94.6 (41.7–138.5)
Total cholesterol (mg/dL)	192 (96–312)
HDL cholesterol (mg/dL)	47 (23–96)
LDL cholesterol (mg/dL)	121 (40–210)
Triglyceride (mg/dL)	127 (42–480)
Plasma glucose (mg/dL)	105 (79–346)
HOMA-IR	3.52 (0.57–40.64)
Hemoglobin A1c (%)	6.2 (3.8–13.2)
Type 2 diabetes mellitus (presence)	78 (40.0%)
Diabetes medication (yes)	64 (82.1%)
Hemoglobin A1c (%)	7.0 (5.2–13.2)
Hypertension (presence)	101 (51.8%)
Smoking (yes)	64 (32.8%)
Max-CIMT (mm)	1.2 (0.4–4.4)
Hyaluronic acid (ng/ml)	54.4 (10.0–3284)
WFA^+^-M2BP (C.O.I.)	0.93 (0.24–8.30)
FIB-4 index	2.17 (0.32–10.82)
NFS	− 0.45 (− 4.52–7.61)
Liver steatosis (1/2/3)	101 (51.8%)/75 (38.5%)/19 (9.7%)
Liver inflammation (0/1/2/3)	9 (4.6%)/110 (56.4%)/69 (35.4%)/7 (3.6%)
Liver ballooning (0/1/2)	39 (20.0%)/129 (66.2%)/27 (13.8%)
NAFLD activity score (≤ 4/ ≥ 5)	135 (69.2%)/60 (30.8%)
Liver fibrosis stage (F0/F1/F2/F3/F4)	29 (14.9%)/62 (31.8%)/43 (22.1%)/49 (25.1%)/12 (6.2%)

Data are presented as numbers or medians (ranges or percentages).

*BMI* body mass index, *AST* aspartate aminotransferase, *ALT* alanine aminotransferase, *γ-GTP* gamma glutamyl transpeptidase, *eGFR* estimated glomerular filtration rate, *HDL* high-density lipoprotein, *LDL* low-density lipoprotein, *HOMA-IR* homeostasis model assessment-insulin resistance, *CIMT* carotid intima-media thickness, *WFA*^+^*-M2BP* Wisteria floribunda agglutinin positive Mac-2-binding protein, *FIB-4* fibrosis-4, *NFS* NAFLD (nonalcoholic fatty liver disease) fibrosis score.

### Correlation between max-CIMT and histopathological severity of NAFLD

**Figure 1:**
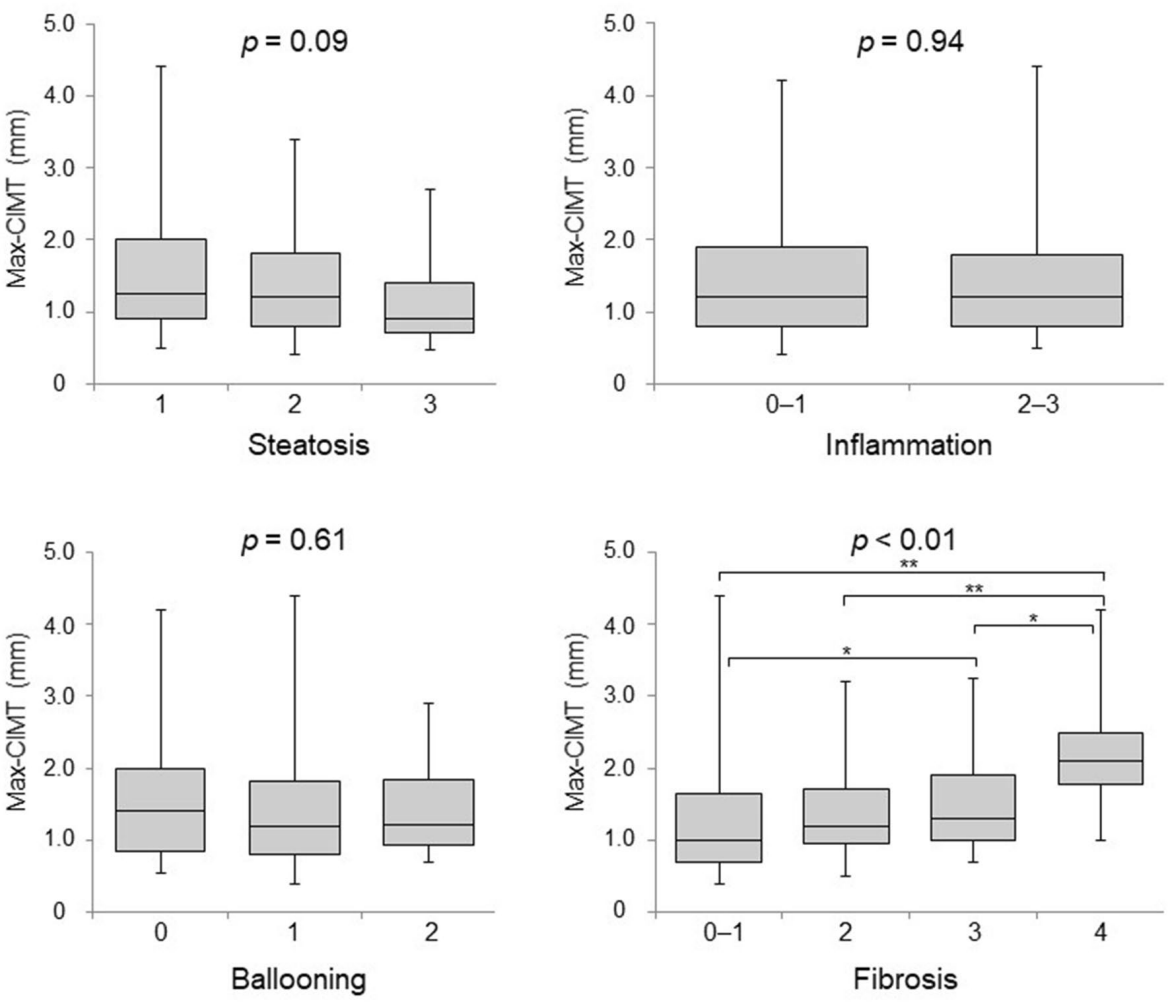
Box and whisker plots of max-CIMT values according to the severity of each histological component in NAFLD patients. CIMT, carotid intima-media thickness; NAFLD, nonalcoholic fatty liver disease. *p < 0.05, **p < 0.01.

**Figure 2:**
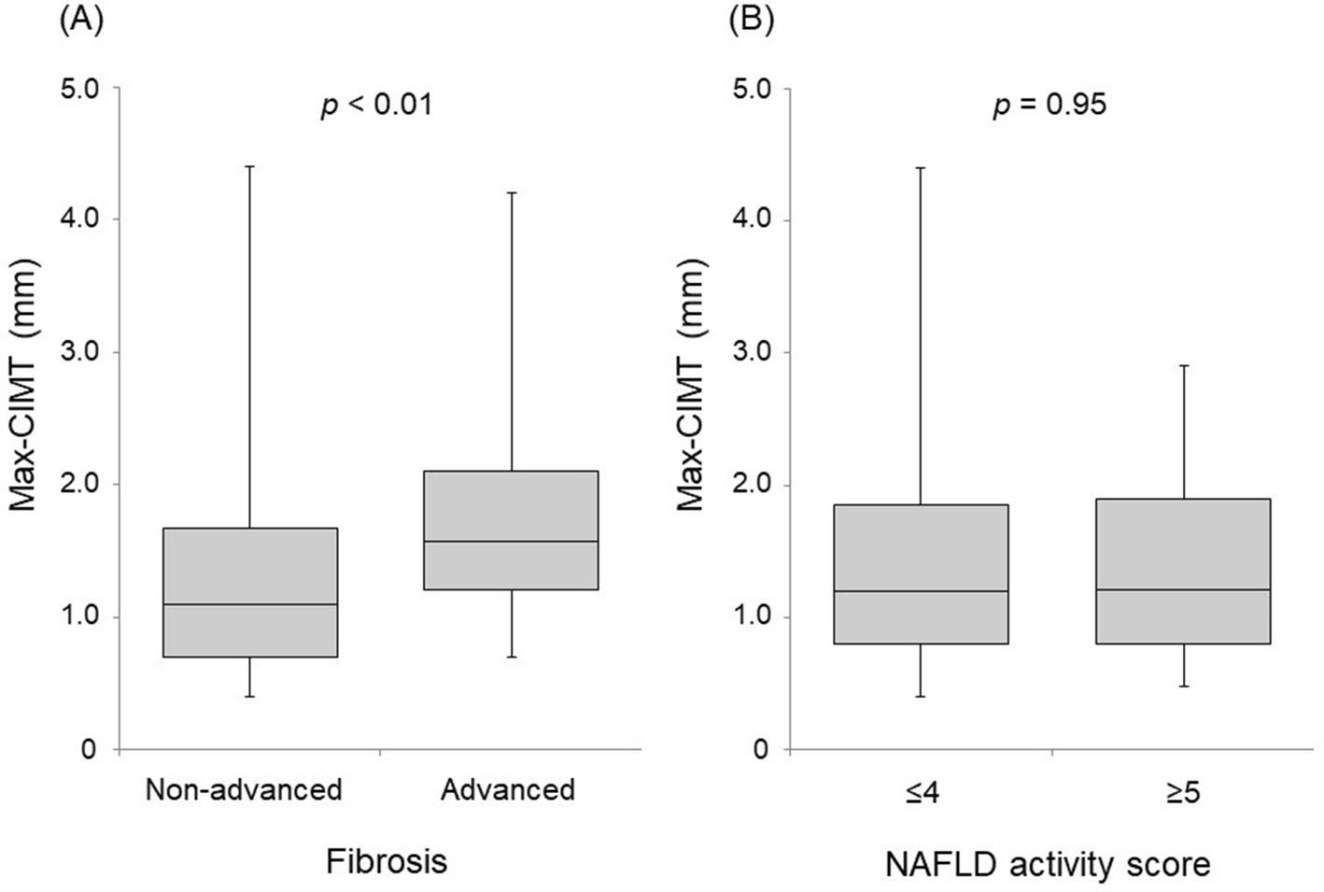
Max-CIMT values in patients with NAFLD according to fibrosis stage (**A**) and NAFLD activity score (**B**). Non-advanced fibrosis: fibrosis stage = F0–2, Advanced fibrosis: fibrosis stage = F3–4; CIMT, carotid intima-media thickness; NAFLD, nonalcoholic fatty liver disease.

As shown in Fig. 1, no correlation with max-CIMT was observed for steatosis (median max-CIMT for grade 1 = 1.3 mm, grade 2 = 1.2 mm, and grade 3 = 0.9 mm; *p* = 0.09), inflammation (median max-CIMT for grade 0–1 = 1.2 mm and grade 2–3 = 1.2 mm; *p* = 0.94), and ballooning (median max-CIMT for grade 0 = 1.4 mm, grade 1 = 1.2 mm, and grade 2 = 1.2 mm; *p* = 0.61). In contrast, max-CIMT significantly differed among the fibrosis stages (*p* < 0.01), and the median values and interquartile ranges increased as the severity of liver fibrosis increased (median max-CIMT for F0–1 = 1.0 mm, F2 = 1.2 mm, F3 = 1.3 mm, and F4 = 2.1 mm). Patients with F3 had significantly higher max-CIMT than those with F0–1 (*p* < 0.05). Of note, patients with F4 had significantly higher max-CIMT than those with F0–1 (*p* < 0.01), F2 (*p* < 0.01), and F3 (*p* < 0.05). Based on their fibrosis stage, the patients were assigned to either non-advanced (F0–2) or advanced (F3–4) fibrosis groups. The max-CIMT was found to be significantly higher in the advanced fibrosis group than in the non-advanced fibrosis group (*p* < 0.01) (Fig. 2A). Regarding the NAFLD activity score (NAS), no significant difference was noted in max-CIMT between the two groups (NAS ≤ 4 versus ≥ 5; *p* = 0.95) (Fig. 2B).

### Factors associated with max-CIMT ≥ 1.2 mm

In the univariate analysis, older age (≥ 65 years), low eGFR (< 60 mL/min/1.73 m^2^), low total cholesterol (< 220 mg/dL), low low-density lipoprotein (LDL) cholesterol (< 140 mg/dL), type 2 diabetes mellitus, hypertension, and advanced fibrosis were significantly associated with max-CIMT ≥ 1.2 mm (Table 2). Multivariate analysis demonstrated that the following four variables were independently associated with max-CIMT ≥ 1.2 mm (Table 2): older age (odds ratio [OR] 5.46; 95% confidence interval [CI] 2.66–11.19; *p* < 0.01), male gender (OR 2.36; 95% CI 1.18–4.73; *p* < 0.05), hypertension (OR 2.24; 95% CI 1.13–4.44; *p* < 0.05), and advanced fibrosis (OR 2.24; 95% CI 1.03–4.86; *p* < 0.05).

**Table 2:** Univariate and multiple logistic regression analyses of factors associated with max-CIMT ≥ 1.2 mm.

Factor	Category	Univariate			Multivariate		
		OR	95% CI	*p* value	OR	95% CI	*p* value
Age	≥ 65 years	6.44	3.34–12.42	< 0.01	5.46	2.66–11.19	< 0.01
Gender	Male	1.73	0.98–3.06	0.06	2.36	1.18–4.73	< 0.05
eGFR	< 60 mL/min/1.73m^2^	2.79	1.12–6.95	< 0.05			
Total cholesterol	< 220 mg/dL	3.29	1.51–7.14	< 0.01			
HDL cholesterol	< 40 mg/dL	1.88	0.96–3.69	0.07			
LDL cholesterol	< 140 mg/dL	3.82	1.91–7.63	< 0.01			
Triglyceride	< 150 mg/dL	1.08	0.60–1.92	0.80			
HOMA-IR	≥ 2.5	167	0.88–3.18	0.12			
Type 2 diabetes mellitus	Presence	2.01	1.12–3.62	< 0.05			
Hypertension	Presence	2.66	1.49–4.75	< 0.01	2.24	1.13–4.44	< 0.05
Smoking	No	1.36	0.74–2.50	0.33			
Liver fibrosis	Advanced fibrosis	3.90	1.98–7.66	< 0.01	2.24	1.03–4.86	< 0.05

*CIMT* carotid intima-media thickness, *OR* odds ratio, *CI* confidence interval, *eGFR* estimated glomerular filtration rate, *HDL* high-density lipoprotein, *LDL* low-density lipoprotein, *HOMA-IR* homeostasis model assessment-insulin resistance.

### Prevalence of max-CIMT ≥ 1.2 mm in relation to liver fibrosis and conventional risk factors

**Figure 3:**
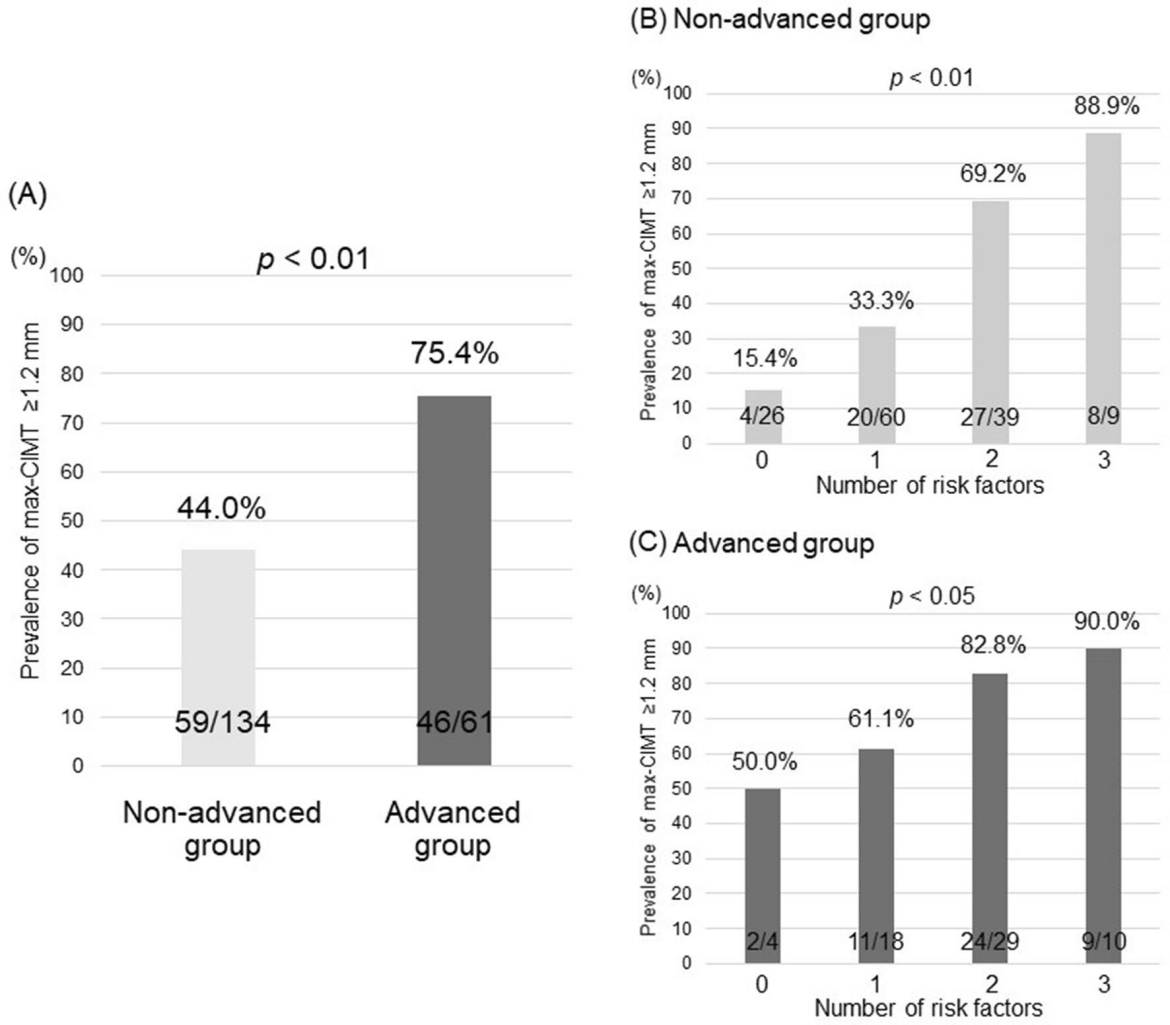
(**A**) The prevalence of max-CIMT ≥ 1.2 mm according to fibrosis stage. The prevalence of max-CIMT ≥ 1.2 mm according to the number of risk factors (older age, male gender, and hypertension) in the non-advanced group (**B**) and the advanced group (**C**). CIMT, carotid intima-media thickness. The non-advance group had fibrosis stage of F0–2, while the advanced fibrosis had fibrosis stage of F3–4.

The prevalence of max-CIMT ≥ 1.2 mm in the advanced fibrosis group (75.4%; 46/61) was significantly higher than that in the non-advanced fibrosis group (44.0%; 59/134) (*p* < 0.01; Fig. 3A). In each group, the prevalence of max-CIMT ≥ 1.2 mm was analyzed based on the number of the aforementioned risk factors (i.e., older age, male gender, and hypertension). In both groups, max-CIMT significantly increased stepwise as the number of risk factors increased (Fig. 3B,C). Intriguingly, in the advanced fibrosis group, even if the number of risk factors was low (0 or 1), the prevalence of max-CIMT ≥ 1.2 mm was 50% or greater. Meanwhile, in the non-advanced fibrosis group, the number of risk factors more strongly affected the prevalence of max-CIMT ≥ 1.2 mm.

### Diagnostic performance of biopsy-based liver fibrosis staging and non-invasive tests for max-CIMT ≥ 1.2 mm

We evaluated the diagnostic performance of biopsy-based liver fibrosis staging and non-invasive tests for max-CIMT ≥ 1.2 mm: the area under the receiver operating characteristics (ROC) curve (AUC) and cutoff values were 0.672 and ≥ F3 for biopsy-based fibrosis staging, 0.668 and 2.06 for FIB-4 index, 0.658 and − 0.674 for NFS, 0.630 and 57.1 ng/ml for hyaluronic acid, and 0.620 and 1.23 C.O.I. for WFA^+^-M2BP, respectively (Supplementary Fig. 2). No significant differences were observed in the AUC between biopsy-based fibrosis staging and non-invasive tests (*p* = 0.90 for FIB-4 index, 0.72 for NFS, 0.35 for hyaluronic acid, and 0.19 for WFA^+^-M2BP). Next, we divided the patients into two groups using the cutoff value corresponding to each fibrosis marker or scoring system and compared the prevalence of max-CIMT ≥ 1.2 mm between the two groups. The prevalence of max-CIMT ≥ 1.2 mm in the high fibrosis marker/scoring system groups was significantly higher than that in the low fibrosis marker/scoring system groups (Supplementary Fig. 3). Finally, we investigated the prevalence of max-CIMT ≥ 1.2 mm according to the number of the aforementioned risk factors in the high and low groups for each fibrosis marker or scoring system. The prevalence of max-CIMT ≥ 1.2 mm significantly increased in a stepwise manner as the number of risk factors increased in both groups for all fibrosis markers and scoring systems (Supplementary Fig. 4). Similar to the results in the biopsy-based fibrosis staging, the prevalence of max-CIMT ≥ 1.2 mm was relatively low in patients with a low number of risk factors (approximately 10% for 0 and around 30% for 1) in the low fibrosis marker/scoring system groups, whereas it was relatively high even in patients with a low number of risk factors (approximately 30%–40% for 0 and 50%–60% for 1) in the high fibrosis marker/scoring system groups. When patients had two or more risk factors, the prevalence of max-CIMT ≥ 1.2 mm was approximately 70%–80% in both high and low fibrosis marker/scoring system groups.

## Discussion

This study demonstrated that advanced liver fibrosis, in addition to conventional risk factors for cardiovascular events (older age, male gender, and hypertension), was significantly and independently associated with a high-risk level of CIMT (surrogate measure of atherosclerosis) in 195 Japanese patients with NAFLD. The meta-analyses and systematic reviews have indicated that there is a positive and significant association between NAFLD and CIMT, irrespective of conventional risk factors^9–11^. Zhang et al. reconfirmed that NAFLD is significantly and independently associated with CIMT in patients with type 1 (but not type 2) diabetes^31^. In the WELCOME study cohort, decreased liver fat (as assessed by magnetic resonance spectroscopy) and reduced necroinflammation (serum cytokeratin-18 level) were found to be associated with suppressed CIMT progression^32^. However, in most of these previous studies, NAFLD was diagnosed by ultrasonography, and not by liver biopsy, which is the gold standard method for diagnosing and staging NAFLD. These data confirmed the association between NAFLD and CIMT, but the role of histological severity of NAFLD and individual histological features requires further studies. Some previous small-scale or pilot studies have reported that 23–57 patients with biopsy-proven NAFLD had significantly higher CIMT than 21–30 control subjects^33–35^. However, due to the limited reports available on the impact of histopathology of NAFLD on CIMT, the consensus on whether the severity of the disease (or a specific histopathological component) could be associated with the CIMT level remains controversial. For instance, in a NAFLD rat model, the hepatic inflammation scores were correlated with CIMT by high-fat diet^36^. To the best of our knowledge, only two studies from Italy have so far reported on how the histopathological severity of liver fibrosis in patients with biopsy-proven NAFLD could be related to carotid atherosclerosis^27,28^. Targher et al. reported that liver steatosis, inflammation, and fibrosis were significantly correlated with CIMT in 85 patients^27^. Petta et al. reported that liver inflammation and fibrosis were associated with carotid artery plaque formation in 162 patients, though not significant in multivariate analysis^28^. However, CIMT values or progression of atherosclerosis may differ according to the racial/ethnic cohorts and lifestyle habits. The CIMT values in Japanese are reportedly lower than those in Caucasian American (even after adjusting for metabolic risk factors)^29^ and Korean populations (although both Japanese and Korean are Asian)^30^. This is the first report to clarify a significant association between the histopathological severity of fibrosis (but not steatosis, inflammation, and ballooning) and the high-risk CIMT level in Japanese patients with biopsy-proven NAFLD.

It is unclear whether the mechanism for liver fibrogenesis affects the progression of atherosclerosis in patients with NAFLD. The association between liver fibrosis and atherosclerosis may be partially explained by a high prevalence of conventional risk factors, such as type 2 diabetes mellitus, dyslipidemia, and hypertension, in NAFLD patients with advanced liver fibrosis^37,38^. In this study, advanced liver fibrosis was significantly associated with higher CIMT values, independent of these risk factors. This suggested the involvement of another unidentified mechanism. Various theories have been proposed for the common mechanism underlying the progression of liver fibrosis and atherosclerosis. Oxidative stress and subclinical inflammation, which play important roles in the progression of NAFLD pathology, induce endothelial dysfunction, which may lead to systemic vascular sclerosis^39,40^. The severity of NAFLD increases the PAI-1 levels, thereby increasing the risk of atherothrombosis^41^. TGF-β, a pro-fibrogenic cytokine, plays a key role in the progression of liver fibrosis and induces arterial intima thickening^42,43^. Various factors are likely involved in complex pathways/networks that could develop and progress both NAFLD and atherosclerosis. Further study is needed to uncover the common or interrelated mechanisms underlying the pathology of NAFLD and atherosclerosis.

In this study, we demonstrated that NAFLD patients with advanced fibrosis had significantly higher max-CIMT values and prevalence of max-CIMT ≥ 1.2 mm compared to those with non-advanced fibrosis. More importantly, advanced fibrosis was a significant and independent factor associated with max-CIMT ≥ 1.2 mm, irrespective of conventional risk factors. The prevalence of max-CIMT ≥ 1.2 mm increased in a stepwise manner with the number of risk factors in both non-advanced and advanced fibrosis groups. Intriguingly, the prevalence of max-CIMT ≥ 1.2 mm was relatively low in patients with non-advanced fibrosis and a low number of risk factors. Thus, attention should be paid to the onset of cardiovascular events, especially when patients with non-advanced fibrosis have two or more conventional risk factors. Meanwhile, patients with advanced fibrosis should be examined for atherosclerosis, irrespective of the absence or number of conventional risk factors.

Although liver biopsy is the gold standard for diagnosis and staging of fibrosis in NAFLD, it offers several limitations, such as the sampling error, cost/effort, and the risk of complications. The FIB-4 index and NFS are highly useful as non-invasive alternatives to liver biopsy^44–46^. In particular, their practical usefulness in patients with NAFLD chiefly includes the ability to exclude advanced fibrosis and avoid liver biopsy^47^. Recently, hyaluronic acid and WFA^+^-M2BP have attracted attention as indicators of early arteriosclerosis as well as markers of liver fibrosis^22–24^. We demonstrated that the diagnostic performance of non-invasive fibrosis markers and scoring systems for max-CIMT ≥ 1.2 mm was similar to that of fibrosis staging via invasive biopsy. Among these non-invasive tests, the FIB-4 index and NFS exhibited relatively high diagnostic performance, presumably because these formulas include age factors that predispose individuals to arteriosclerosis. In fact, FIB4-index and NFS have been reported to be important predictors of cardiovascular mortality, which is the main cause of death in patients with NAFLD^48,49^. Specifically, patients with advanced liver fibrosis should be closely monitored for the development of arteriosclerosis and the risk of cardiovascular events, as well as liver function itself and liver-related complications.

In this study, type 2 diabetes mellitus, a critical risk factor for cardiovascular events^50,51^, was not identified as an independent factor associated with CIMT in multivariate analysis. Considering that treatment for type 2 diabetes mellitus improves CIMT^52–55^, possible explanations for the lack of independent association include variations in the duration and severity of type 2 diabetes mellitus and diabetes treatment, such as diet, exercise, and drug therapy. A part of the diabetic patients in this study cohort was treated by diabetologists shortly after the diagnosis of diabetes. Most of them had been receiving diabetes medication and were experiencing good control in terms of their hemoglobin A1c levels. Therefore, the results of this study should be verified in a larger NAFLD cohort or the general population.

This study has several limitations. First, this was a retrospective and hospital-based study, which involves a potential for patient selection bias. Second, the number of patients was relatively small, especially those with advanced fibrosis. A large cohort including many patients with advanced fibrosis is required to verify the association between severe atherosclerosis and more advanced fibrosis stages. Third, as aforementioned, the community-based general population (serving as a control) was not taken into consideration, suggesting that the influence of risk factors, including type 2 diabetes mellitus, on CIMT was not accurately evaluated in patients with NAFLD.

In conclusion, this study demonstrated that advanced fibrosis, as well as conventional risk factors (older age, male gender, and hypertension), is significantly and independently associated with the high-risk CIMT level for cardiovascular events in Japanese patients with biopsy-proven NAFLD. Non-invasive fibrosis markers and scoring systems could be helpful when estimating the risk of atherosclerosis progression in patients with NAFLD. Specifically, patients with NAFLD accompanied by advanced live fibrosis should be examined and monitored for atherosclerosis and the risk of cardiovascular events.

## Methods

### Patients

Among the patients who presented to Nippon Medical School Hospital, Nippon Medical School Chiba Hokusoh Hospital, and Shinmatsudo Central General Hospital between July 2011 and December 2020, 357 patients with suspected NAFLD underwent liver biopsy. Among the 357 patients, 195 who met the inclusion criteria, did not fill the exclusion criteria, and underwent carotid artery ultrasonography within 3 months of liver biopsy were enrolled in this study (Supplementary Fig. 1). The inclusion criteria were as follows: (1) age ≥ 18 years; (2) undergoing liver biopsy with consent; and (3) the diagnosis of NAFLD based on histopathological evaluation according to the guidelines of the European Association for the Study of the Liver^56–58^. The exclusion criteria were as follows: (1) daily alcohol consumption ≥ 30 g for men and ≥ 20 g for women; (2) other chronic liver diseases, such as viral hepatitis B or C, autoimmune hepatitis, Wilson disease, or hemochromatosis; and (3) secondary causes of steatosis, including use of drugs (e.g., amiodarone and tamoxifen), total parenteral nutrition, and inborn errors related to metabolism. A careful interview and clinical, laboratory, and imaging examinations were conducted at the time of liver biopsy in all the patients.

The study protocol complied with the ethical guidelines established following the 2013 Declaration of Helsinki. The Ethics Committee of Nippon Medical School Chiba Hokusoh Hospital approved this study (approval number, 603). All patients provided written informed consent before their participation.

### Clinical evaluation and laboratory data

Clinical evaluations and laboratory data were collected at the time of liver biopsy. Body mass index (BMI) was calculated as weight (kg) divided by the square of height (m^2^). Blood pressure was measured in a seated position, at least twice and several minutes apart, and the mean value was calculated and utilized for analysis. Hypertension was diagnosed as systolic blood pressure ≥ 135 mmHg or diastolic blood pressure ≥ 85 mmHg, or when the patients were being treated with an antihypertensive drug^59^. Dyslipidemia was diagnosed when the total cholesterol level was ≥ 220 mg/dL**,** high-density lipoprotein (HDL) cholesterol level was < 40 mg/dL, triglycerides level was ≥ 150 mg/dL or LDL cholesterol level was ≥ 140 mg/dL, or when the patients were being treated with an antihyperlipidemic drug^60^. Type 2 diabetes mellitus was diagnosed with reference to the 2006 World Health Organization criteria or when the patients reported having been receiving treatment with an oral hypoglycemic agent and/or insulin.

Laboratory evaluation included complete blood count, routine liver (aspartate aminotransferase, alanine aminotransferase [ALT], total bilirubin, albumin, alkaline phosphatase, and gamma-glutamyl transpeptidase) and renal (urea nitrogen, creatinine, and estimated glomerular filtration rate [eGFR]) biochemistry tests, fasting lipid profiles (total cholesterol, triglycerides, HDL cholesterol, and LDL cholesterol), and diabetes-related tests (fasting plasma glucose, hemoglobin A1c, and immunoreactive insulin). As an index of insulin resistance, the homeostasis model assessment-insulin resistance (HOMA-IR) was calculated as follows: fasting immunoreactive insulin (μU/mL) × fasting plasma glucose (mg/dL)/405^61^. Hyaluronic acid^18^ and WFA^+^-M2BP^19–21^, both of which have been reported as liver fibrosis markers in NAFLD, were also determined. In addition, the FIB-4 index and NFS were calculated to estimate the degree of liver fibrosis, as reported elsewhere^44–46^.

### Carotid ultrasound

Carotid artery ultrasonography was conducted using a B-mode ultrasound system with a 7.5-MHz linear transducer within 3 months of liver biopsy. According to the guidelines of the Japan Society of Ultrasonics in Medicine^62^, all scans were performed by skilled laboratory technicians who were blinded to the patient information. The examination included the near and far walls of the common carotid arteries (CCAs), carotid bulbs (CBs), and internal carotid arteries (ICAs). The thickest point, including plaque lesions in the entire scanned intra-media thickness of the CCAs, CBs, and ICAs, was defined as max-CIMT. With reference to a report on the general Japanese papulation, patients with max-CIMT ≥ 1.2 mm were considered to be at a high risk for cardiovascular events^63^.

### Histopathological evaluation

Of the patients with suspected NAFLD based on ultrasound and increased ALT levels, some consented to liver biopsy, which is the gold standard for diagnosis and staging of fibrosis in patients with NAFLD. Histopathological evaluation was conducted by at least two experienced pathologists from each facility who were blinded to the patient’s data. In case of a disagreement related to the diagnosis, the pathologists discussed until they reached a consensus. NAFLD was diagnosed when the lipid droplet deposition was observed in ≥ 5% of hepatocytes. Steatosis, lobular inflammation, ballooning, and liver fibrosis were semi-quantitatively evaluated according to the NASH Clinical Research Network scoring system^64^. Steatosis was graded as 0–3 based on the percent of hepatocytes with steatosis on biopsy specimens (0: < 5%, 1: 5–33%, 2: 33–66%, 3: > 66%). Lobular inflammation was graded as 0–3 based on the number of inflammatory foci per 200 × field (0: no foci, 1: < 2 foci, 2: 2–4 foci, 3: > 4 foci). Ballooning was graded as 0–2 based on the number of hepatocytes with this change (0: none, 1: few cells, 2: many cells or prominent ballooning). The NAS was considered as the unweighted sum of steatosis, lobular inflammation, and ballooning scores. NAS of ≥ 5 was strongly correlated with the diagnosis of NASH^64^. The stage of fibrosis was classified as follows: F0 = no fibrosis, F1 = perisinusoidal or periportal fibrosis, F2 = perisinusoidal and portal/periportal fibrosis, F3 = bridging fibrosis, and F4 = cirrhosis. F3–4 was provisionally designated as advanced fibrosis.

### Statistical analysis

Continuous variables are presented as medians and ranges. Categorical variables were presented as numbers and percentages. Continuous variables with skewed distributions were compared among or between the groups using the Kruskal–Wallis test or the Mann–Whitney *U*-test, respectively. The Steel–Dwass test was performed when the Kruskal–Wallis test indicated a significant difference among the groups. Categorical variables were compared using Fisher’s exact test. The variables associated with max-CIMT ≥ 1.2 mm on univariate analysis (probability threshold, *p* < 0.10) were included in multiple logistic regression. The Cochran–Armitage trend test was employed to evaluate increases in the prevalence of max-CIMT ≥ 1.2 mm in relation to increases in the number of risk factors, such as older age, male gender, and hypertension. An ROC curve was generated to determine the values of non-invasive markers and scoring systems that most rationally predicted max-CIMT ≥ 1.2 mm. The cutoff value for each non-invasive test was determined corresponding to the point of the ROC curve, which was closest to the upper-left corner of the plot (i.e. point [0, 1]). The DeLong’s test was applied to compare the AUC values of non-invasive fibrosis markers and scoring systems with those of biopsy-based fibrosis staging. All statistical analyses were performed using the IBM SPSS version 17.0 (IBM Japan, Tokyo, Japan). The level of statistical significance was set at *p* < 0.05.

## Supplementary Information

Supplementary Figures.

## Data Availability

All data generated or analyzed during this study are included in this published article.
