# Supplementary material for: Liver fibrosis is associated with carotid atherosclerosis in patients with liver biopsy-proven nonalcoholic fatty liver disease
Source: Sci Rep. 2021 Aug 5;11:15938. doi: 10.1038/s41598-021-95581-8 (PMC8342487; doi:10.1038/s41598-021-95581-8)
Supplement: Supplementary file 1 — Supplementary Figures. [file 41598_2021_95581_MOESM1_ESM.pdf]

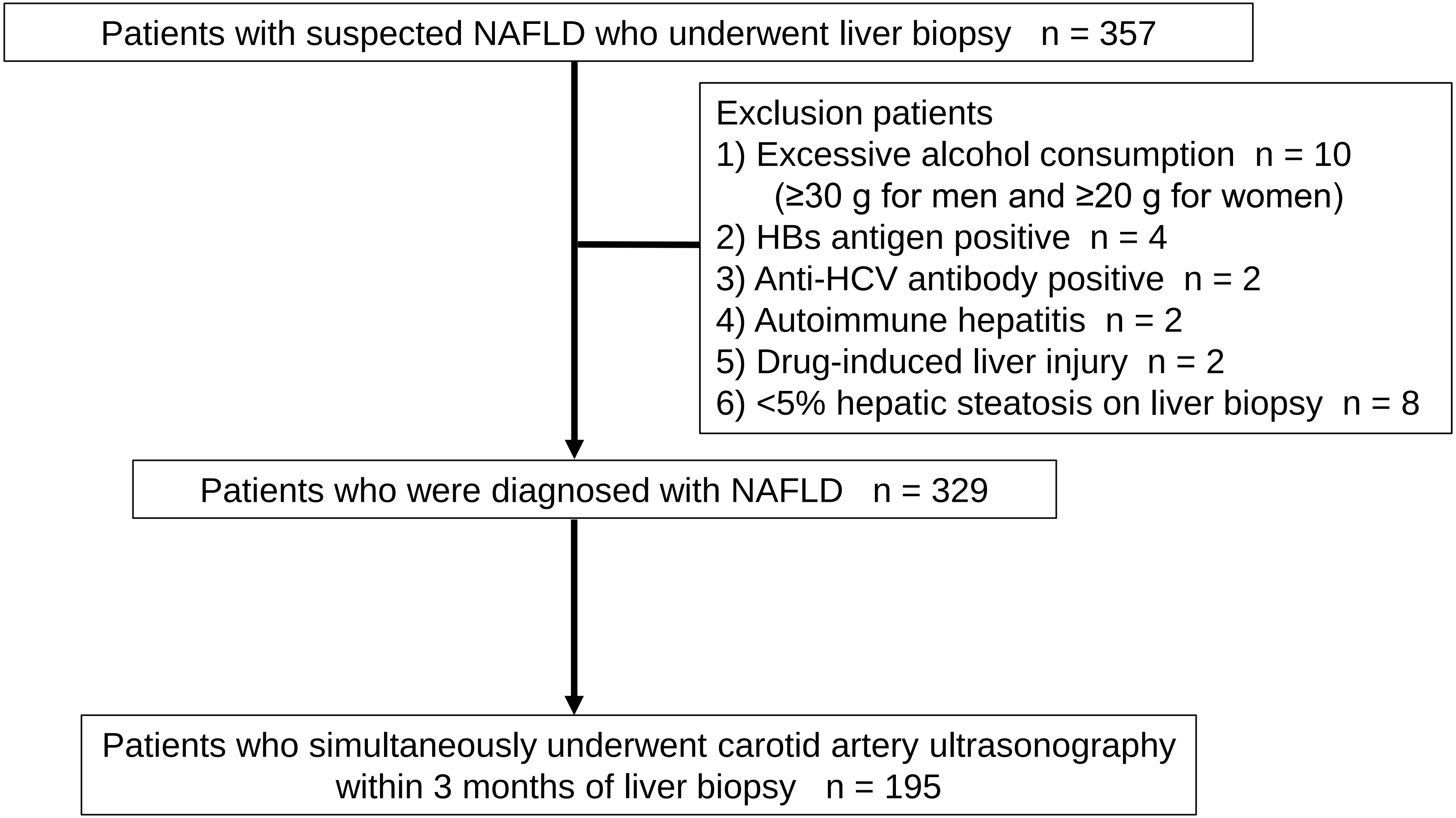


Supplementary figure 1.

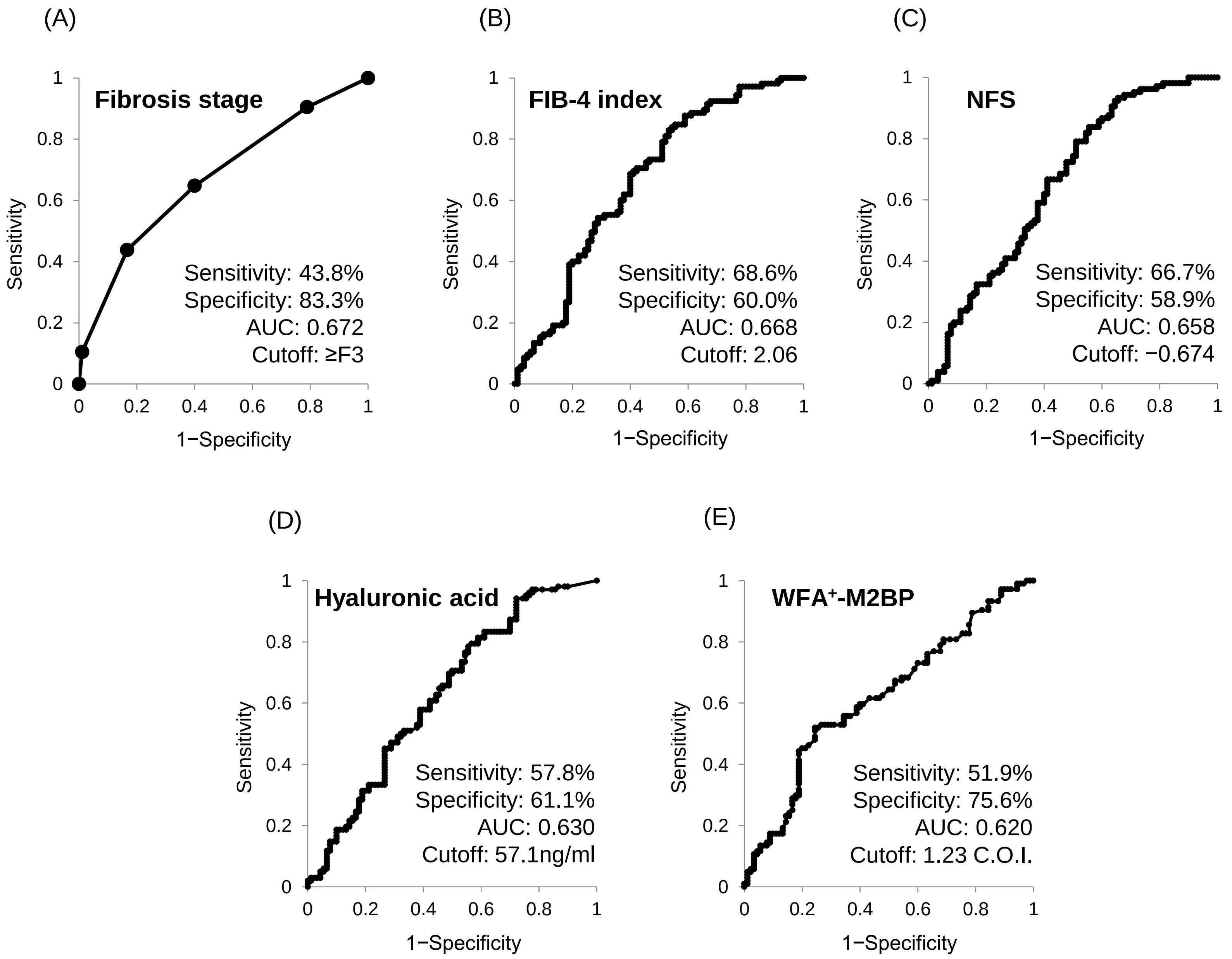


Supplementary figure 2.

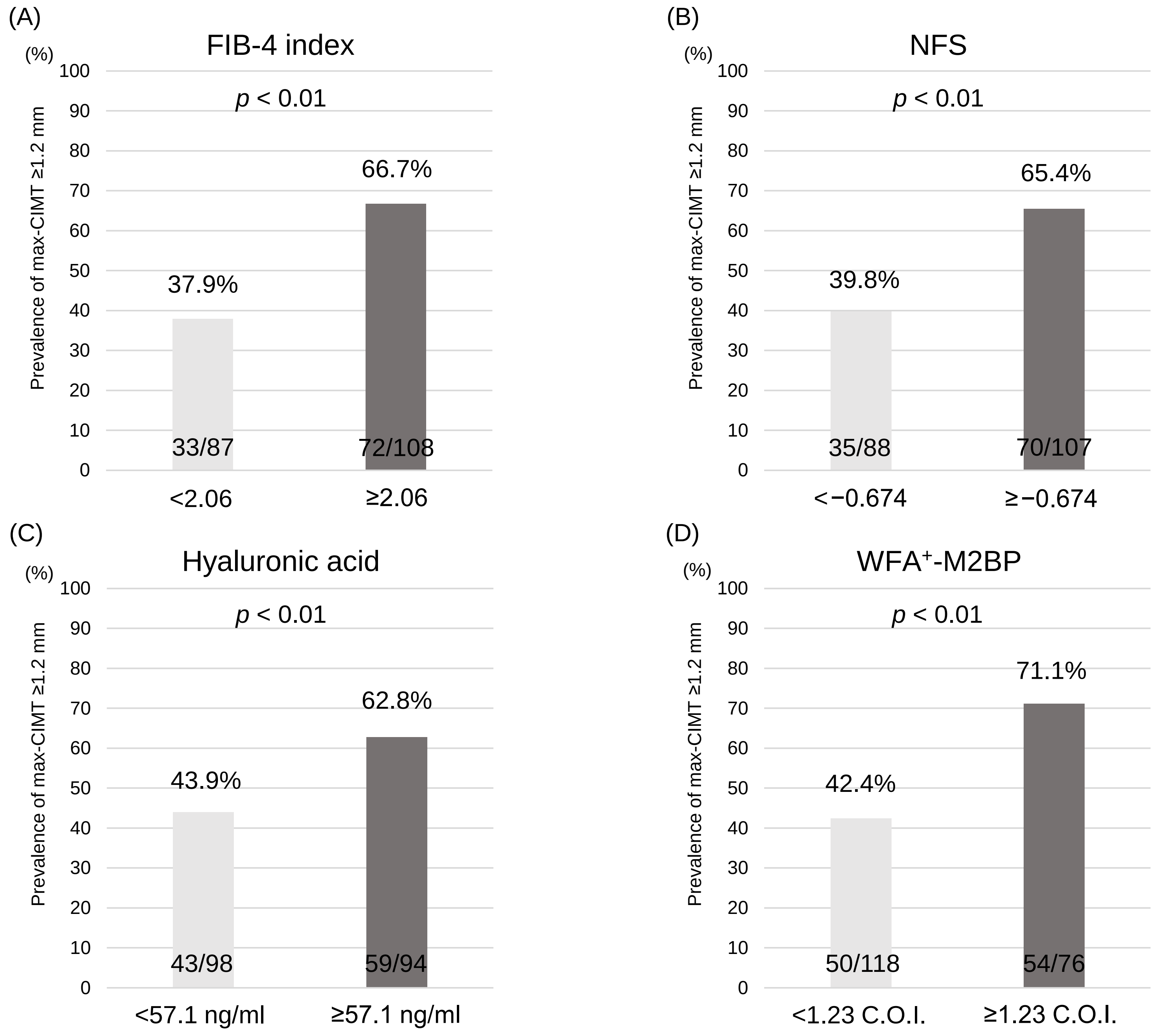


Supplementary figure 3.

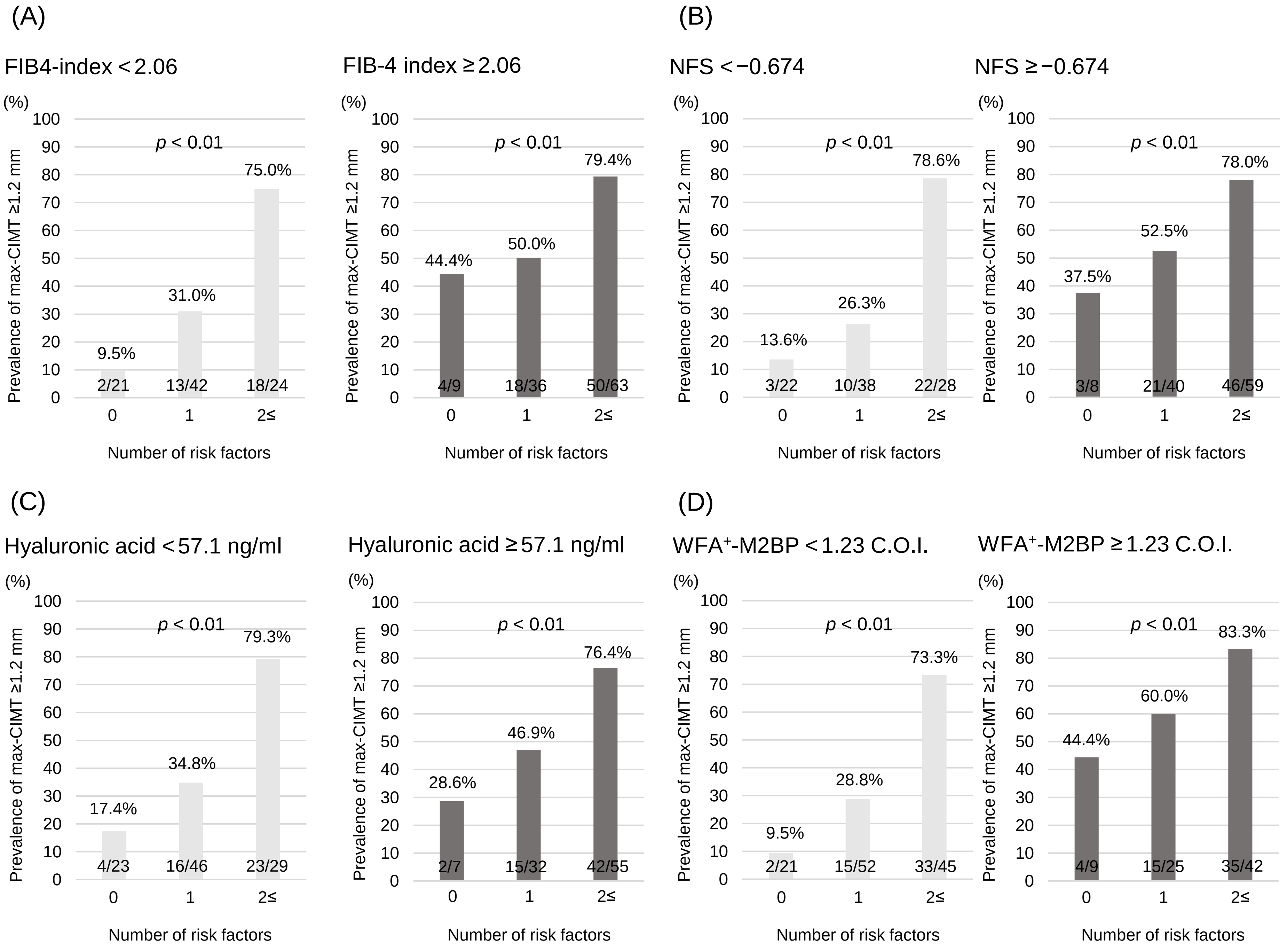


Supplementary figure 4.

Supplementary figure 1. Flow chart for patient inclusion. NAFLD, nonalcoholic fatty liver disease.

Supplementary figure 2. Receiver-operating characteristic curves of fibrosis stage (A), FIB-4 index (B), NFS (C), hyaluronic acid (D), and WFA$^{+}$-M2BP (E) for predicting max-CIMT ≥1.2 mm. CIMT, carotid intima-media thickness; AUC, area under the curve; FIB-4, fibrosis-4; NFS, NAFLD (nonalcoholic fatty liver disease) fibrosis score; WFA$^{+}$-M2BP, Wisteria floribunda agglutinin positive Mac-2-binding protein; C.O.I., cutoff index.

Supplementary figure 3. The prevalence of max-CIMT ≥1.2 mm according to the cutoff values of FIB-4 index (A), NFS (B), hyaluronic acid (C), and WFA$^{+}$-M2BP (D). CIMT, carotid intima-media thickness; FIB-4, fibrosis-4; NFS, NAFLD (nonalcoholic fatty liver disease) fibrosis score; WFA$^{+}$-M2BP, Wisteria floribunda agglutinin positive Mac-2-binding protein; C.O.I., cutoff index.

Supplementary figure 4. The prevalence of max-CIMT ≥1.2 mm according to the number of risk factors (older age, male gender, and hypertension) in the subgroups, which were divided by the cutoff values of FIB-4 index (A), NFS (B), hyaluronic acid (C), and WFA$^{+}$-M2BP (D). CIMT, carotid intima-media thickness; FIB-4, fibrosis-4; NFS, NAFLD (nonalcoholic fatty liver disease) fibrosis score; WFA$^{+}$-M2BP, Wisteria floribunda agglutinin positive Mac-2-binding protein; C.O.I., cutoff index.
